# Recent Advances in Developing Inhibitors for Hypoxia-Inducible Factor Prolyl Hydroxylases and Their Therapeutic Implications

**DOI:** 10.3390/molecules201119717

**Published:** 2015-11-19

**Authors:** So Yeon Kim, Eun Gyeong Yang

**Affiliations:** 1 Center for Theragnosis, Biomedical Research Institute, Korea Institute of Science and Technology, Hwarangno 14-gil 5, Seongbuk-gu, Seoul 136-791, Korea; soyeonkim@kist.re.kr; 2 Department of Biomedical Engineering, Korea University of Science and Technology (UST), KIST campus, Seoul 136-791, Korea; 3 Department of Biological Chemistry, Korea University of Science and Technology (UST), KIST campus, Seoul 136-791, Korea

**Keywords:** prolyl hydroxylase (PHD) inhibitor, hypoxia-inducible factor (HIF), structure-based drug design (SBDD), high-throughput screening (HTS)

## Abstract

Hypoxia-inducible factor (HIF) prolyl hydroxylases (PHDs) are members of the 2-oxoglutarate dependent non-heme iron dioxygenases. Due to their physiological roles in regulation of HIF-1α stability, many efforts have been focused on searching for selective PHD inhibitors to control HIF-1α levels for therapeutic applications. In this review, we first describe the structure of PHD2 as a molecular basis for structure-based drug design (SBDD) and various experimental methods developed for measuring PHD activity. We further discuss the current status of the development of PHD inhibitors enabled by combining SBDD approaches with high-throughput screening. Finally, we highlight the clinical implications of small molecule PHD inhibitors.

## 1. Introduction

The ability to maintain oxygen homeostasis is essential to the survival of aerobic species. Since the discovery of hypoxia-inducible factor (HIF)-1 [1], signaling mechanisms underlying oxygen-sensing by a HIF transcription factor have been extensively studied in biological contexts. HIFs, composed of oxygen-labile α and constitutively expressed β subunits, drive the transcription of numerous genes involved in diverse cellular processes including erythropoiesis, angiogenesis, energy metabolism, ischemia, and inflammation [2].

HIF-α has been shown to exist in three different isoforms, among which two HIF-α isoforms, HIF-1α and HIF-2α, likely regulate different sets of genes, although their downstream genes largely overlap [3]. While HIF-1β remains stable regardless of oxygen availability, the stability of HIF-α is sensitive to the oxygen level. The mechanism by which oxygen controls HIF-1α has been revealed by the identification of HIF prolyl hydroxylases (PHDs) [4]. Under normoxia, PHD hydroxylates proline residues in the oxygen dependent degradation (ODD) domain of HIF-1α, thereby promoting its binding to von Hippel Lindau protein (pVHL)-elonginB-elonginC (VBC), leading to active ubiquitination and degradation with a half-life of approximately 5 min [5,6,7,8]. On the other hand, the oxygen deprivation under hypoxia impairs hydroxylation of HIF-1α by PHDs, resulting in reduced HIF-1α turnover and subsequent induction of target gene transcription.

PHDs belong to the family of the dioxygenase enzymes that require oxygen, iron, and 2-oxyglutarate (2-OG) for their catalytic activity. Their low affinity to oxygen, which is about 2 to 10 times higher than physiological oxygen concentrations, enables the enzymes to act as oxygen sensors [9]. Three PHD isoforms (PHD1, PHD2, and PHD3) have been identified, and their substrates are known to be quite diverse and isoform-specific. PHD1 controls the expression level of nuclear factor kappa-light-chain-enhancer of activated B cells (NF-κB) by hydroxylation-mediated inactivation of the inhibitor of NF-κB kinase β [10]. In addition, PHD1 is involved in cell proliferation by degrading a cell cycle regulator Cyclin D1 in a hydroxylase activity-dependent manner [11]. The large subunit of RNA polymerase II Rbp1 has been shown to be another substrate for PHD1, and prolyl hydroxylation is required for its VHL-dependent degradation. On the other hand, PHD3 plays major roles in neural development, immune system function, cell migration and apoptosis [12]. Activating transcription factor 4 and the human homolog of the *C. elegans* biological clock protein CLK-2 have been identified as non-HIF substrates for PHD3 [13,14]. Non-muscle actin is also hydroxylated at proline residues by PHD3, and inhibition of PHD3 activity leads to increased filamentous F-actin assembly [15]. Distinctly from its hydroxylase activity, PHD3 can act as a scaffolding protein and regulate various signaling pathways [16,17].

**Figure 1:**
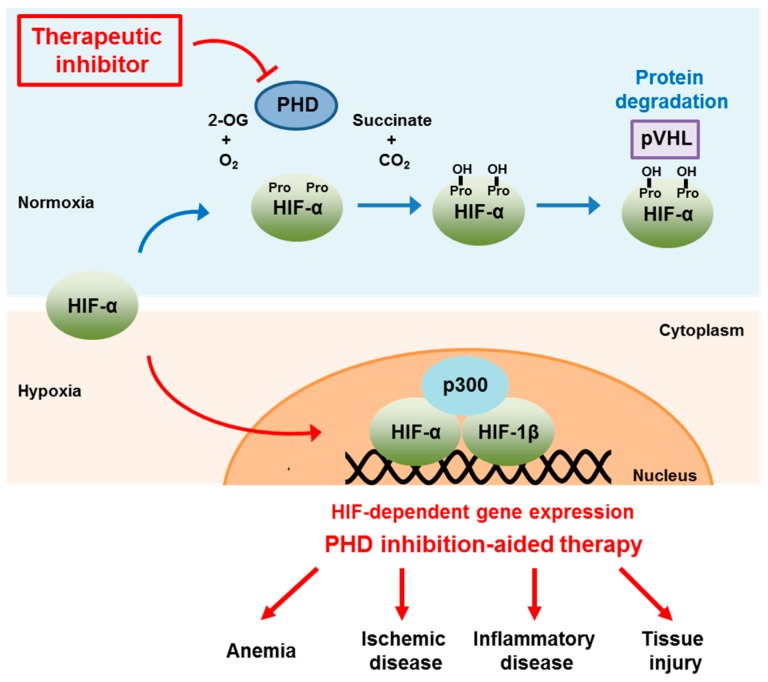
Schematic diagram illustrating HIF-dependent gene expression by inhibiting PHD activity and its therapeutic implications.

PHD2 is considered critical in regulating the HIF pathway, although its hydroxylase activity is necessary for regulating other signaling pathways including cofilin phosphorylation and the NDRG3 protein degradation [18,19]. Specifically, enhanced angiogenesis, and increased levels of vascular endothelial growth factor (VEGF)-A and erythropoietin (EPO) were observed in conditional knockout of PHD2 [20,21,22]. Such observations, along with the previous report that HIF enhanced EPO release and concomitantly increased erythropoiesis [1,23], imply that activation of HIF by modulating PHDs could be beneficial for patients with anemia and ischemia-related diseases. Accordingly, pharmacological approaches to manipulate the HIF pathway by inhibiting PHD activity have been pursued to treat systemic and local hypoxia-related diseases (Figure 1).

The PHD structure has contributed to the efficient design of structure-based PHD inhibitors by reducing time and cost, while the advancement of fast and reliable methods of determining PHD activity has permitted the rapid identification of inhibitors based on high-throughput screening (HTS). Although some PHD inhibitors are currently in clinical trials, potential side effects of targeting HIF via PHD inhibition should be taken into consideration, because both aberrant HIF activation and PHD knockdown are linked to the development of other diseases such as cancer and erythrocytosis [24,25].

## 2. PHD2 Structure

After the first crystal structure of the C-terminal catalytic domain of the human PHD2 complexed with a 2-OG-competitive isoquinoline inhibitor was reported by McDonough *et al.* [26], 13 additional crystal structures of PHD2 with either inhibitors or HIF-derived peptides have been reported in the PubMed database. In this section, we briefly describe the structural differences between PHD2 in the absence and presence of the HIF substrate. Guided by these structural differences, numerous researchers have designed inhibitors that block the active site or are small mimetics of 2-OG without chelating iron. Detailed information about these structure-based inhibitors will be provided in Section 4.

PHD2 crystallizes as a homotrimer, while it exists as a monomer in solution. In the catalytic domain of PHD2, eight β strands create double stranded β helix walls around the active site. Outside the β helix walls, there are three α helices that hold the β helix walls in place. Compared to other 2-OG dependent oxygenases, PHD2 has a relatively deep pocket with a narrower opening to the active site. The 2-OG pocket of PHD2 consists of hydrophobic residues including Ile256, Met299, Ala301, Tyr303, Tyr310, Thr325, Ile327, Tyr329, Leu343, Phe366, Val376, Ala365, Thr387 and Trp389. On the other hand, the catalytic triad residues necessary for iron binding are conserved (His313-Asp315-His374) similar to other 2-OG dependent oxygenases. Importantly, mutagenesis of Arg383 to Ala causes the complete loss of catalytic activity, suggesting that Arg383 plays a crucial role in cofactor binding within the active site.

The crystal structure of the catalytic domain of PHD2 with the C-terminal ODD domain of HIF-1α provided additional information about the conformational changes associated with substrate binding [27]. As expected from the sequence of the HIF-1α peptide, its interaction with PHD2 is highly hydrophobic. Furthermore, the conformational changes induced by HIF-1α binding enable the buried metal center to be exposed to oxygen. These additional conformational changes can be blocked by bulky heterocyclic inhibitors, which stabilize the closed conformation. In comparison, small molecule inhibitors likely hamper the formation of the iron-oxygen complex, rather than HIF-1α binding.

## 3. Assays for Measuring PHD Activity

PHD activity assays can be classified based on their detection methods (Table 1). Except for those using radioactive isotopes as starting materials, most assays employ either the interaction between hydroxylated HIF-1α and VBC or mass spectrometry (MS), in which a product of the enzyme reaction can be directly analyzed. While these assays are routinely performed in cell-free conditions with purified proteins, monitoring of PHD activities in the cellular context is executed using a variety of cell-based assays.

Although not classified in this section, novel methods for measuring PHD activities are continuously being developed. For example, an oxygen consumption assay has been reported to measure oxygen level changes induced by active PHDs using a fiber optic oxygen sensor system [28]. By directing the transcription of galactosidase following the interaction between hydroxylated-HIF-α and VHL, the degree of HIF-α hydroxylation by active PHDs has been determined based on its proportionality to the increase in blue staining from the galactosidase-induced hydrolysis of X-gal [29]. Luciferase activity elicited by the interaction between bead-attached HIF and luciferase-ligated VHL has also been exploited to measure PHD activity [30].

**Table 1:** Summary of PHD activity assays.

Method	Description	Advantage	Disadvantage	Ref.
Radioactive isotope-based	Detection of reaction products using [^14^C]-labeled 2-OG Detection of interactions between VHL and HIF-1α, either of which is labeled with [^35^S]-methionine	Highly sensitive	Use of radioactive materials Can be non-specific	[4,31,32,33]
Fluorescence-based	Detection of fluorescent derivatives of 2-OG Detection of increased FP of HIF-1α peptides upon binding to VHL Detection of TR-FRET by interactions between VHL and HIF-1α Detection of electrochemi-luminescence by interaction between VHL and HIF-1α	Suitable for HTS Easy to perform	Can be non-specific Cannot be used for fluorescent inhibitors	[34,35,36,37]
MS-based	Detection of reaction products using [^14^C]-labeled 2-OG Detection of interactions between HIF and PHD using [^35^S]-methionine labeled proteins Detection of inhibitors after dissociation from PHDing	Highly sensitive	Difficult for HTS Can be time-consuming	[38,39,40]
Cell-based	Detection of ODD domain-mediated luciferase activity	Physiological environment	May not be appropriate for bulky molecules Can be non-specific, and hard to interpret	[41]

### 3.1. Radioactive Isotope-Based Assays

Most commonly used 2-OG dependent oxygenase assays involve the capture and detection of ^14^CO_2_ gas resulting from oxidative decarboxylation of [1-^14^C]-labeled 2-OG [4,31]. Depending on the location of the radiolabel on 2-OG, [^14^C]-labeled succinate rather than ^14^CO_2_ can be produced [42]. Considering inefficient trapping of ^14^CO_2_, either [^35^S]-methionine-labeled HIF-1α or [^35^S]-methionine-labeled VHL instead of radiolabeled 2-OG has been used to measure PHD activity by monitoring the interaction between HIF-1α and VHL after hydroxylation reactions [43]. Similarly, an assay based on the interaction of the hydroxylated HIF peptide with VHL has been reported and later adapted for HTS [32,33]. In this scintillation proximity assay, streptavidin-coated beads are used to capture the biotin-labeled HIF peptide bound to [^35^S]-methionine-labeled VHL. On the other hand, nuclear magnetic resonance (NMR)-based detection has been utilized with [1,2,3,4-^14^C_4_]-labeled 2-OG as a reporter ligand [44]. The displacement of 2-OG following the binding of a competitive ligand was successfully probed by measuring the decrease in the NMR intensity.

### 3.2. Fluorescence-Based Assays

Despite their sensitivity, radioactive isotope-based assays present safety and disposal problems, and involve cumbersome washing and detection steps. Such drawbacks have thus prompted development of assays employing fluorescence technologies, including fluorescence intensity, fluorescence polarization (FP), and fluorescence resonance energy transfer (FRET). In addition, fluorescence-based detection applied in enzyme assay formats facilitates assay transformations into HTS formats.

McNeill *et al*., reported a fluorescence-based assay for 2-OG dependent hydroxylases based on the conversion of unreacted 2-OG into a fluorescent derivative via a reaction with *ortho*-phenylenediamine (OPD) [34]. In addition, this assay was used to screen proline analog inhibitors for PHD3 activity [45]. Although the method is simple and easily adaptable to HTS, it can be problematic in testing small molecules with functional groups reactive with OPD or having fluorescence properties similar to the fluorescent derivatives.

FP that monitors molecular movement and rotation was also adopted in the PHD activity assay first reported by Cho *et al.* [46]. In this assay, the increase in FP from the fluorescein-labeled HIF-1α ODD peptide is directly proportional to the activity of PHD, since only the hydroxylated HIF-1α peptide by active PHDs can bind to the VBC complex. The FP-based assay was further applied to screening of a collection of 1040 biologically active compounds [47], and some potent inhibitors such as baicalein and clioquinol analogs were identified [47,48,49]. Moreover, the inhibitory effects of several peptide derivatives with modifications at the Pro564 have been explored [35,36]. The specificity conferred by the sequence of the HIF-1α peptide renders these peptide inhibitors selective toward PHDs without inhibiting the HIF asparagine hydroxylase, factor inhibiting HIF (FIH) [36].

The FRET assay employs “donor” and “acceptor” fluorophores in proximity, and time-resolved (TR)-FRET occurring between europium and red fluorophores such as allophycocyanin (APC), Cy5, and Alexa 647 has been widely used to develop assays for kinases and nuclear receptors [50]. Such principle was exploited for a PHD2 activity assay in which FRET between the europylated VBC complex and the APC-labeled HIF peptide was measured [37]. When the APC-labeled HIF peptide is hydroxylated by PHD2, the hydroxylated peptide binds to europylated VBC, thereby enhancing FRET efficiency. This sensitive and facile assay could be readily applicable to a large HTS of 60,000 compounds per day. In addition to the TR-FRET-based assay, an electrochemiluminescence (ECL) assay was also described by using a ruthenium chelated tris-bipyridiyl ligand attached to VBC [37]. Streptavidin coated paramagnetic beads were used to capture the hydroxylated HIF peptide bound to VBC via the biotin-streptavidin interaction, and the fluorescence produced by ruthenium after applying current was quantified. Although the ECL assay showed a greater dynamic range and a higher signal to background (20-fold selectivity) than the TR-FRET assay did, no further study using the ECL assay has yet been reported.

### 3.3. MS-Based Assays

Various MS-based methods have been developed for identification of PHD inhibitors. A combination of limited proteolysis with the matrix-assisted laser desorption/ionization (MALDI)-MS method has been applied in screening for PHD inhibitors that alter protein structure [38]. The extent of which the proteolytic stability of PHD2 changes in the presence of inhibitors is compared to that for the native PHD2 with 2-OG and iron. A small library of metal chelators was screened for PHD inhibitory effects by analyzing the ratio of the unmodified HIF-1α peptide peak to the hydroxylated peak with a mass shift of +16 obtained from MALDI-time of flight (TOF) mass spectra [39]. On the other hand, Vachal *et al.* developed a high-throughput affinity selection MS (AS-MS), which enabled the screening of approximately 500,000 molecules for PHD2 inhibitors [40]. After incubation with hundreds of compounds, PHD2-inhibitor complexes were first separated from unbound PHD2 using a size-exclusion column, followed by MS analysis of the compounds dissociated from PHD2.

### 3.4. Cell-Based Assays

Most assays described above utilize purified PHD proteins in a cell-free system, where the physiological environment of enzyme may not be properly reflected. For example, the endogenous 2-OG concentration is higher in cells than typically used *in vitro*, which could lead to a dramatic decrease in cellular PHD inhibitory as well as HIF activating potency of 2-OG competitive inhibitors [33]. Furthermore, some bulky inhibitors such as peptide-based inhibitors may not freely penetrate the cell membrane. Therefore, cell-based assay systems are indispensable for evaluation of the inhibitor efficacy in the physiological context to facilitate drug discovery. Using a luciferase reporter system fused to an HIF-ODD, a cell-based assay was developed for HTS with more than 85,000 compounds, resulting in more than 160 confirmed hits [41]. Given the contribution of all PHD isoforms to hydroxylation of the HIF-ODD substrate, inhibitors detected by cell-based assays may well be non-specific toward PHDs. In addition, some of the detected inhibitors could be simple divalent metal ion chelators, instead of acting at the specific active site of an enzyme, because the structure modifications by structure-activity relationship (SAR) rarely affected the inhibition potency [41].

## 4. Development of PHD Inhibitors

SBDD has emerged as a major strategy for drug development due to its advantages of time and cost efficiency. Briefly, the process for SBDD is as follows: first, an active site of the target protein should be identified, and once it is well-defined, a drug candidate compound library is prepared either by virtual HTS or by fragment-based *de novo* design. Subsequently, the docking score of each compound is determined, and highly ranked compounds are finally synthesized for further testing. Following the first determination of the crystal structure of PHD2 [26], researchers have made attempts to develop PHD inhibitors based on SBDD. Meanwhile, drug discovery based on HTS requires highly specific, simple, and easily executable enzyme activity assays that are amenable to automation. As described in Section 3, various PHD enzyme assays using fluorescence or cell-based methods permit the screening of ~100,000 compounds per day for drug candidates. The identification of new inhibitors using HTS methods can be further utilized as the basis for a drug candidate compound library for either SBDD or SAR assays.

### 4.1. SBDD for PHD Inhibitors

The first PHD inhibitor drug candidates obtained using SBDD came from the research activities of Proctor & Gamble. Warshakoon *et al.*, reported the inhibitory potencies of four different series of compounds that are derivatives of 8-hydroxyquinoline-7-carboxamide, pyridine Gly amide, imidazo[1,2-*a*]pyridine, and pyrazolopyridines, all of which blocked the active site of PHD2 and enhanced VEGF production *in vitro* [51,52,53,54]. In addition, introducing benzyloxymethyl groups at the pyridine C5 position was found to strengthen the interaction with the Tyr 310, Arg 322, Met 299, and Gln239 residues of the hydrophobic pocket (compound **1**, Table 2) [53]. Furthermore, the addition of a hydroxyl group at C3 of the pyridine ring, similar to the isoquinoline series of PHD inhibitors, significantly increased the potency of inhibitors with a half-maximal inhibitory concentration (IC_50_) of 0.017 μM (compound **2**, Table 2), which was recently demonstrated using dynamic combinatorial MS with SAR [55].

**Table 2:** PHD inhibitors developed by SBDD.

Compound Number	Compound Structure	IC_50_	Producer
**1**	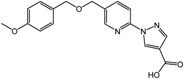	2.4 μM	Proctor & Gamble
**2**	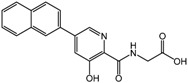	0.017 μM	Proctor & Gamble
**3** (TM6008)	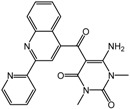	N/A	Tokai University
**4** (TM6089)	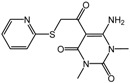	N/A	Tokai University
**5** (JNJ-42041935)	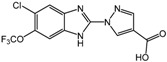	0.1 μM	Janssen
**6**	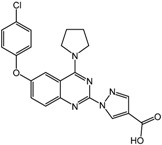	0.05 μM	Janssen
**7**	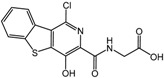	<25 μM	Crystal-Genomics
**8**	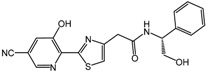	2.1 μM	Crystal-Genomics
**9**	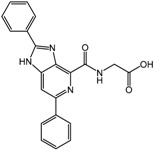	0.003 μM	Amgen

Researchers at Tokai University in Japan used docking simulation with the PHD2 structure (PDB:2HBT) to identify novel PHD inhibitors TM6008 and TM 6089 (compounds **3** and **4**, Table 2) [56], whose structures were selected based on a previously reported inhibitor FG-0041 [57]. Interestingly, while TM6089 does not share an iron chelating motif and is devoid of iron chelating activity, TM6008 acts as an inhibitor with this motif. The anti-inflammatory effect of TM6008 mediated by PHD inactivation was further demonstrated [58]. TM6008 has been shown to be efficacious in the treatment of inflammatory diseases, because PHD2 inhibition by TM6008 suppressed tumor necrosis factor-α mediated inflammation in macrophages [58]. A recent study further implicated TM6008 in enhancing cell survival after hypoxia by suppressing the protein expression of PHD2 and p53 [59].

The Janssen company also exploited SBDD methods to develop PHD inhibitors with a series of benzimidazole-2-glycinecarboxamides [60,61]. The co-crystal structure of the inhibitor candidate with PHD2 suggested that Arg383 and Tyr303 at the active site of PHD were important for inhibitor binding [62]. Non-planar chelation with the active site iron was also energetically favorable, and the resulting 2-(1*H*-pyrazol-1-yl)-1*H*-benzimidazole analogs showed improved potencies both *in vitro* with purified enzyme and in whole cell EPO release assays (compound **5**, JNJ-42041935 in Table 2) [60,61]. Furthermore, oral administration of JNJ-42041935 exhibited significant hematopoietic effects in animal models. It should be noted that JNJ-42041935 inhibits all PHD isoforms non-selectively, which contributes to the stimulation of hepatic EPO production [51] because only the triple knockout of all PHD isoforms is capable of inducing hepatic EPO synthesis [63]. Among additional compound series such as quinazolinones and aminoquinazolinones, quinazolinones exhibited unexpected high potency with IC_50_ values of sub nM and ~60% stimulation of EPO release in HEP3B cells. One of the aminoquinazolinone derivatives, compound **6** (Table 2) showed a relatively high IC_50_ value of 50 nM compared to other derivatives, but a 96% stimulation of EPO in cells [64].

CrystalGenomics in the Republic of Korea reported active site PHD inhibitors based on the crystal structure of PHD2 with 2-OG [62]. The first compound in the series was benzothienopyridine Gly amide as shown in Table 2 (compound **7**). Although its IC_50_ value was not clearly specified, the company claimed that it enhanced EPO release by more than 50-fold relative to normal levels, with enhanced VEGF release in cell culture [65]. The introduction of a nitrile group in the 5-position of the pyridine ring provided an additional interaction with Tyr329 and Arg383, and substantially increased the inhibition potency. SAR analysis of several synthesized compounds led to the development of [2-(3-hydroxypyridin-2-yl)-thiazol-4-yl]-acetamide derivatives, such as compound **8** (Table 2). Despite its IC_50_ value *in vitro* similar to those of the other derivatives, compound **8** showed more than a 40-fold enhanced EPO production with good physicochemical properties [66].

The Amgen research group made efforts to develop PHD2 inhibitors employing various approaches in combination with both SBDD and HTS methods. Inhibitors identified using HTS methods will be described in the next section. SBDD based on the crystal structure of PHD2 with a 4-hydroxyisoquinoline inhibitor resulted in the azabenzimidazole scaffold [67]. Among 25 compounds tested, compound **9** (Table 2) was found to have an IC_50_ value of 3 nM. Furthermore, molecular modeling predicted that a carbonyl group and nitrogen in the pyridine ring are involved in its iron chelation, and the distance between these two atoms is crucial for activity.

### 4.2. PHD Inhibitors Developed by HTS Methods

A recent review by Rabinowitz described discovery efforts based on HTS with various methods in detail and provided information on the small molecule PHD inhibitors [68]. Although some other assays described in Section 3 could be used in principle, numerous clinical candidates have been discovered using a TR-FRET-based PHD assay as an HTS method. Many drug companies such as GlaxoSmithKline (GSK), Amgen, Merck, and Bayer in particular have relied on TR-FRET detection in HTS for PHD inhibitors. GSK started with the 4-hydroxy-2-quinolone derivatives to discover their selective inhibition of PHD3 over PHD2 [69]. The introduction of an aminocarbonyl group into pyrimidone and pyridazone analogs resulted in compound **10** (Table 3), which showed improved cellular potency with an EPO half-maximal effective dose of 1–20 nM [70]. Further HTS of their compound collections revealed a series of quinazoline-2,4-diones and 4-oxo-2-thioxo-7-quinazolines devoid of carboxylate groups for iron chelation [71]. For these compounds, an N atom in the heterocycle and either a keto or thiono group from the core appear to be involved in their binding to the active site.

Employing a TR-FRET method, Amgen reported PHD selective inhibitors (IC_50_ of 65 nM for PHD2) over collagen prolyl hydroxylases (CPH) 1 and CPH2 (IC_50_ of > 40,000 nM for CPH1) [72]. In addition, N-hydroyxythiazoles such as compound **11** (Table 3) were discovered, and subsequent SAR and modeling analysis suggested that the acetic acid side chain, in addition to the sulfone group, is essential for enzyme potency. Merck also utilized a TR-FRET assay in guiding SAR development [73,74,75]. For example, compound **12** (Table 3) showed an IC_50_ of 0.7 nM, and SAR development revealed that the nitrogen-containing heterocycle at the C2 position of the central pyrimidine ring is important for its potency. On the other hand, the 96-well plate FRET assay was used to produce several patents granted to Bayer on dihydropyrazolones, thiazolines, dihydropyrazolethiones, and dihydropyrazolone compound series such as compound **13** (Table 3), which showed IC_50_ values in the range of 70–760 nM (compound **13**, IC_50_ of 70 nM) [76,77].

**Table 3:** PHD inhibitors developed by HTS.

Compound Number	Compound Structure	IC_50_	Producer
**10**	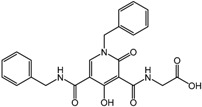	EC_50_ for EPO 1–20 nM	GSK
**11**	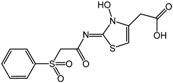	N/A	Amgen
**12**	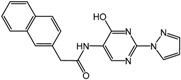	0.7 nM	Merck
**13**	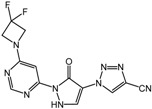	70 nM	Bayer
**14**	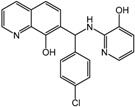	2 μM	Cornell University
**15**	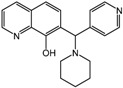	10 μM	Cornell University
**16**	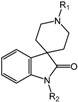	4 nM	Merck
**17**	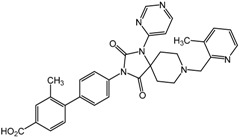	0.2 nM	Merck

Reporter cell lines that constitutively express ODD-luciferase were used by Smirnova *et al*., for cell-based HTS, and catechol and branched oxyquinoline derivatives among 295 hits were chosen for further biological studies [41]. Their results indicate that the structural adjustment of the inhibitor to ensure a proper fit in the active site rather than its iron chelation ability plays a crucial role in reporter activation. Compound **14** (Table 3) upregulated HIF-1α and its target genes including EPO, VEGF, LDHA and PGK1. Furthermore, it protected cortical neurons against oxidative stress in homocysteic acid cellular model. In contrast, compound **15** (Table 3), a good iron chelator (K_Fe_ ~0.08 μM), did not exhibit such effects. Theriault *et al*., also applied a cell-based assay with the luciferase readout to identify compounds to stabilize HIF-1α [78]. After screening more than 300,000 compounds, secondary imaging assays of the HIF-1α nuclear translocation led to the identification of N-([1,1′-biphenyl]-4-yl-methyl)-6-phenyl-3-(2-pyridinyl)-1,2,4-triazin-5-amine (ML228). This compound has potent metal chelation activity in solution, but unlikely binds directly to the PHD enzyme to exert specific inhibition.

Vachal *et al*., at Merck used high-throughput AS-MS for PHD2 inhibitor selection to identify spiroindole derivatives among the 500,000 tested molecules as new candidates [40]. Throughout the initial SAR optimization, they found optimal structures exhibiting increased inhibition potencies, resulting in production of compound **16** (Table 3). However, upon further evaluation by *in vivo* pharmacodynamic screening assays, they showed suboptimal pharmacokinetics (PK) and off-target activity. After subsequent structural adjustment and optimization processes to enhance the PK profile and *in vivo* efficacy, as well as physical and chemical properties, compound **17** (Table 3) was chosen as a promising candidate for preclinical testing.

## 5. Therapeutic Implications

### 5.1. Anemia

Although iron repletion and administration EPO analogs are currently used to treat anemia, several issues have emerged, such as high cost of EPO analogs and associated resistance as well as side effects. In this regard, the most obvious therapeutic implication of PHD inhibitors is that they can be used to treat anemia by enhancing EPO secretion via upregulation of HIF-1α. In addition, various downstream genes activated by HIF-1α could be beneficial as well. Several review papers thoroughly cover the recent advances in the development of PHD inhibitors targeting anemia [79,80]. Current clinical trials are described in Table 4.

**Table 4:** Examples of current clinical trials targeting PHDs.

Drug	Patient Population (Clinical Trial Phase)	Purpose of Study	Clinical Trials. Gov. Number (Status)
FG-4592	Subjects with anemia associated with chronic kidney disease without dialysis (phase III)	Evaluate the efficacy for treatment of anemia correction and hemoglobin correction as well as their maintenance	NCT01750190 (recruiting)
AKB-6548	Subjects with end stage renal disease undergoing chronic hemodialysis (phase II)	Evaluate the hemoglobin response (efficacy), safety, and tolerability	NCT02260193 (activated, but not recruiting)
GSK-1278863	Hemodialysis dependent subjects with anemia associated with chronic kidney diseases, chronically hyporesponsive to recombinant human EPO (phase II)	Evaluate the safety and efficacy after switching from recombinant human EPO	NCT02075463 (recruiting)
BAY-85-3934	Subjects with anemia associated with chronic kidney disease on dialysis, in USA and Japan (phase II)	Evaluate the efficacy and safety of oral BAY-85-3934 and active comparator in the long term treatment	NCT02064426 (recruiting)

### 5.2. Ischemic Disease

In ischemic injury, it has been shown that both initial restriction of oxygen and blood supply by ischemia and reperfusion by rapid restoration of oxygen can damage the tissue [81], because fast reoxygenation is usually associated with a profound inflammatory response. Regarding the role of HIF in ischemic disease, three different pathways have been revealed. First, the HIF signaling pathway is linked to the ischemic preconditioning process. When myocardial tissue is pre-exposed to short, non-lethal ischemia, the injured tissue region is significantly reduced. HIF has been shown to be involved in this preconditioning process, and its stability is important for this effect [82]. Secondly, the interaction between HIF and the circadian rhythm protein period 2 (PER2) is critical for glycolytic enzyme production of the ischemic heart, which is necessary for metabolic adjustment during hypoxia [83]. Lastly, constitutively active HIF in remote ischemic preconditioning enhances plasma interleukin-10 transcription, leading to a concomitant decrease of myocardial infarct size [84].

The potential of PHD inhibitors for protecting neurons and repairing the brain after stroke was highlighted by Karuppagounder and Ratan [85]. Initial studies with deferoxamine (DFO) treatment focused on its role as an iron chelator for HIF stabilization, followed by its target gene expression as a mechanism for stroke neuroprotection. However, recent experimental data from different research groups suggested that direct inhibition of PHDs by small molecule inhibitors rather than HIF stability plays a role in neuroprotection [86,87,88]. Nevertheless, it should be noted that the PHD inhibitors used in these studies are iron chelators such as DFO, ethyl 3,4-dihydroxybenzoic acid and dimethyloxaloylglycine (DMOG), but not isotype-specific PHD inhibitors, and therefore other possibilities such as inhibition of other pathways should be considered.

### 5.3. Inflammatory Disease

Inflamed tissues are usually characterized by inflammatory hypoxia owing to increased metabolism and low levels of oxygen and glucose in the inflamed region [89,90,91,92]. In fact, HIF mediated pathways are closely involved in numerous human diseases including inflammatory bowel disease, Crohn’s disease, acute lung injury, and infectious diseases [89,90]. Oxygen deprivation under hypoxia and pro-inflammatory molecules can stabilize HIF-1α in the inflamed region, and hypoxia *per se* can be an inflammatory stimulus by enhancing pro-inflammatory cytokines. In contrast, stabilized HIF-1α can promote anti-inflammation via molecules such as adenosine and netrin-1, as well as tissue repair through reduced cellular apoptosis. Furthermore, a recent work by Scholtz [93] demonstrated the role of PHD1 and FIH in regulating pro-inflammation, suggesting that a hydroxylase inhibitor can be used to dampen excessive inflammation. In addition to these findings, PHD inhibitors such as DMOG and FG-4497 were shown to significantly improve intestinal inflammation [94,95].

### 5.4. Tissue Injury

Because wound healing involves various processes including inflammation, angiogenesis, and vasculogenesis, which are all regulated by HIF-1α target genes, the stabilization of HIF-1α by PHD inhibition can be used to effectively treat tissue injuries and wounds. In accordance with this speculation, several studies showed the possibility of using PHD inhibitors to improve wound healing in diabetic mice [96,97,98,99,100]. A recent review by Ruthenborg *et al*., provided detailed information about the role of HIF-1α in pathogenic wound repair (e.g., diabetic wounds) and its therapeutic potentials [101].

## 6. Perspectives

Significant efforts have been made to discover PHD inhibitors using a variety of methods including chemoinformatics-based (either ligand-based or structure-based) drug screening, combinatorial library production, and HTS. As a result, four discovered compounds are currently in human clinical trials (Table 4). However, some of the major deleterious effects caused by PHD inhibition are a cause for concern and remain to be solved. The most significant issue is HIF stabilization, because the elevation of the HIF-α level is often observed in numerous human diseases like tumors [102] and some of its downstream molecules such as VEGF can be a target for inhibition in some diseases. In contrast, several studies showed evidence that PHD inhibition can inhibit tumor growth and invasiveness [103,104], illustrating that the role of HIF in cancer is highly complex. Therefore, tissue-specific and isoform-selective PHD inhibitors are necessary to target specific disease states with avoiding unwanted effects. Tissue-specific delivery of PHDs appears to be achievable based on the route of administration. For example, based on the study of HIF-dependent lung protection [105], it is conceivable that HIF can be selectively stabilized in alveolar epithelial cells when PHD inhibitors are inhaled. Considering its PK profile and rate of clearance, the structure of an inhibitor can be optimized further for selective tissue exposure. Since the expression and function of individual PHDs have been shown to vary, isoform-selective PHD inhibitors are desirable. Obtaining crystal structures of PHD isoforms might also be helpful in refining their active sites and conformational differences. Lastly, the distinct roles of HIF-α isoforms should be considered, because inhibition of specific PHDs could give rise to differential responses of HIF-1α and HIF-2α [3].
